# Second-hand smoke and chronic bronchitis in Taiwanese women: a health-care based study

**DOI:** 10.1186/1471-2458-10-44

**Published:** 2010-01-28

**Authors:** Chia-Fang Wu, Nan-Hsiung Feng, Inn-Wen Chong, Kuen-Yuh Wu, Chien-Hung Lee, Jhi-Jhu Hwang, Chia-Tsuan Huang, Chung-Ying Lee, Shao-Ting Chou, David C Christiani, Ming-Tsang Wu

**Affiliations:** 1 Graduate Institute of Occupational Safety and Health, Kaohsiung Medical University, Kaohsiung, Taiwan; 2 Chest Medicine, Medical Department, Kaohsiung Armed Forces General Hospital, Kaohsiung, Taiwan; 3 Division of Pulmonology, Department of Internal Medicine, Kaohsiung Medical University, Kaohsiung, Taiwan; 4 Division of Environmental Health and Occupational Medicine, National Health Research Institutes, Miaoli, Taiwan; 5 School of Public Health, Kaohsiung Medical University, Kaohsiung, Taiwan; 6 Department of Family Medicine, Kaohsiung Medical University Hospital, Kaohsiung, Taiwan; 7 Department of Environmental Health, Harvard School of Public Health, Boston, MA, USA; 8 Department of Occupational Medicine, Kaohsiung Municipal Hsiao-Kang Hospital, Kaohsiung, Taiwan; 9 Center of Excellence for Environmental Medicine, Kaohsiung Medical University, Kaohsiung, Taiwan

## Abstract

**Background:**

Cigarette smoking cannot fully explain the epidemiologic characteristics of chronic obstructive pulmonary disease (COPD) in women, particularly for those who rarely smoke, but COPD risk is not less than men. The aim of our study is to investigate the relationship between second-hand smoke (SHS) exposure and chronic bronchitis in Taiwanese women.

**Methods:**

We used Taiwan's National Health Insurance Bureau claims data in 1999, and cross-checked using criteria set by the American Thoracic Society; there were 33 women with chronic bronchitis, 182 with probable chronic bronchitis, and 205 with no chronic bronchitis during our interview time between 2000 and 2005. We measured second-hand smoke (SHS) exposure by self-reported measures (household users and duration of exposure), and validated this by measuring urinary cotinine levels of a subset subjects. Classification of chronic bronchitis was also based on spirometry defined according to the GOLD guidelines to get the severity of COPD.

**Results:**

Women who smoked and women who had been exposed to a lifetime of SHS were 24.81-fold (95% CI: 5.78-106.38) and 3.65-fold (95% CI: 1.19-11.26) more likely to have chronic bronchitis, respectively, than those who had not been exposed to SHS. In addition, there was a significant increasing trend between the severity of COPD and exposure years of SHS (*p *< 0.01). The population attributable risk percentages of chronic bronchitis for smokers and those exposed to SHS were 23.2 and 47.3% respectively.

**Conclusions:**

These findings indicate that, besides cigarette smoking, exposure to SHS is a major risk factor for chronic bronchitis in Taiwanese women.

## Background

Chronic obstructive pulmonary disease (COPD), a chronically progressive disease that includes chronic bronchitis and emphysema [1], remains a major public health problem and its prevalence and mortality are increasing throughout the world [2-4]. In Taiwan, COPD has ranked stably as the 10^th^-12^th ^leading cause of death since 2000. However, until now, COPD is still the second most prevalent reason for visits to physicians (8,135 and 6,408 per 100,000 persons in 2000 and 2006 respectively) and its medical care cost is also highest at the 6^th ^rank [5] among leading causes of death. Furthermore, COPD is estimated to be the third most common cause of death worldwide by 2020 [3]. Since no effective treatment exists to block its progress [6] and taking into account the economic burden of medical care resources worldwide [7], prevention of COPD is critical to public health.

Cigarette smoking is a well-known risk factor for COPD in men [8]. The prevalence of cigarette smoking in Taiwanese men is very high (55-60%), but low among Taiwanese women (3-4%) from 1974 to date [9-11], suggesting that potentially, a large number of non-smoking Taiwanese women are exposed to second-hand smoke (SHS). According to a government report, COPD ranked as both the 12^th ^leading cause of death in men (5.9 per 100,000) and women (3.6 per 100,000) in 2008 [5]. Liu and his colleagues also found 81% deaths of COPD in never smokers in rural Chinese women with the same low smoking rates as Taiwanese women [12]. All these results suggest that cigarette smoking per se cannot fully explain the epidemiologic characteristics of COPD in Taiwanese women, particularly those who rarely smoke but have COPD risk not less than men.

Similar to cigarette smoke, besides carcinogenic components, SHS contains many potent pulmonary irritants [13], all known to cause inflammatory or irritant reactions in the airways leading to respiratory symptoms or lung function impairment [14,15]. Recently, Woodruff and his colleagues provided a biological link between SHS exposure and the development of inflammatory processes and even COPD in mice experiments due to alveolar macrophage recruitment and activation [16]. In 2002, Jaakkola and Jaakkola reported that there was still limited evidence of SHS effect on the risk of COPD [14]. Although a series of studies was published subsequently [17-28], the findings were conflicting. Thus, we conducted a health-care-based case-control study to investigate the association between SHS exposure and risk for chronic bronchitis in Taiwanese women, whose smoking rate was very low.

## Methods

### Study area and population

Our study setting was Kaohsiung City, a harbor city located on the southwestern coast of Taiwan (153.6 km^2 ^and has 11 administrative districts), belonging administratively to Taiwan's National Health Insurance Program Kao-Ping District, which administers to 3,234,941 persons in Taiwan's Kaohsiung and Pingtung areas in 1999. During that year, 1,065,624 Kaohsiung City residents had applied for health insurance compensation. Ninety-six percent of Taiwan's citizens are enrolled in Taiwan's National Health Insurance Scheme, and 93% of the physicians there have contracts with the National Health Insurance Bureau [29], making its database nationally representative.

According to the Kao-Ping District insurance database for 1999, a total of 221,965 female Kaohsiung residents 40 years of age or older made health insurance claims. Kaohsiung municipal records confirmed that they had lived in the city for 5 years or more. These women were defined as study cases if they had received a diagnosis of definite or suspected chronic bronchitis (ICD-9 code: 491) at least twice in 1999. Those who had sought medical attention for traffic accidents (ICD-9 code: E800-E848) or acute gastroenteritis (ICD-9 code: 008.8; 009.1; 558.3; 558.9) in the same year and had never been diagnosed as having chronic bronchitis were used as study controls. We excluded both study cases and controls if they had been diagnosed with other pulmonary-associated diseases, including asthma (ICD-9 code: 493), pulmonary tuberculosis (ICD-9 code: 011.9 or 010-018), bronchiectasis (ICD-9 code: 494), fibrotic cyst (ICD-9 code: 277), pulmonary tumor (ICD-9 code:162), emphysema (ICD-9 code: 492), extrinsic allergic alveolitis (ICD-9 code: 495), or chronic airway obstruction, not elsewhere classified (ICD-9 code: 496). We were left with 1,846 study cases and 4,624 controls (Additional file 1).

Between June, 2000 and March, 2005, about one-third of the study cases (n = 622) were randomly selected, interviewed and administered pulmonary function tests in their homes. Once a study case was successfully recruited, interviewed and administered a pulmonary function test, we found one study control age-matched to within 3 years. The matched control was chosen from the same administrative area to adjust for the possible influence from external environmental hazards such as air pollution from large factories or traffic. After excluding those who refused to participate or those we could not locate, etc., we had 210 study cases and 210 controls successfully complete the interview and pulmonary function tests, making a response rate of 67.3% and 68.4% respectively. The average age (±SD) (years) of responders (n = 210) and non-responder study cases were 64.6 ± 9.6 and 63.2 ± 10.8, which was not significantly different (*p *= 0.11); of responders and non-responder study controls were 64.6 ± 9.7 and 64.7 ± 11.2, which was also not significantly different (*p *= 0.91). This study was approved by IRB at Kaohsiung Medical University, and all potential subjects signed informed consent forms.

### SHS exposure measurement

#### Questionnaire

Epidemiologic data was obtained by trained interviewers who conducted personal interviews in the homes of our study subjects. The questionnaire, which was a modified version of the American Thoracic Society Division of Lung Disease Respiratory Symptom Questionnaire [1], included the demographic characteristics and socioeconomic status of the participants and questions about the presence of chronic bronchitis symptoms, family history of respiratory diseases, and exposure to airborne household chemicals, including cigarette smoking, SHS, cooking oil fumes, and incense burning. The interview lasted around 30 minutes.

Detailed information about cigarette smoking and SHS exposure was collected concerning their three age periods: childhood (≤ 20 years) exposure at home, early adult life (20-40 years) and late adult life (> 40 years) exposure at home and the workplace. Smokers were defined as those who responded "Yes" to the question, "Have you ever smoked, on average, more than one cigarette per day for at least one year?" during any of their three age periods. Ex-smokers were defined as those who had quit at least one year during any of their three age periods; we categorized them as smokers due to the small sample size.

If the study subject was not a smoker, two separate self-reported measures were used for SHS exposure assessment. The first measure was based on household's or co-workers' cigarette smoke used separately, and second-hand smokers were defined as those who responded "Yes" to the question, "Have you ever lived with a smoker or co-workers smoking nearby while indoors working and, on average, been exposed face-to-face to more than one cigarette per day for at least one year?" during any of the three age periods. The second measure was based on duration of SHS exposure in lifetime exposure with a series of detailed questions including what year exposure started and what year exposure ceased or how long they had been exposed (in years) to SHS. We calculated lifetime cumulative SHS exposure by summing the number of years the second-hand smokers reported exposure to smokers in the three age periods at home and work separately and combined. However, only 33% of women in our study had worked during their early adult life and most of them became housewives during their later adult life, so the level of cumulative exposure was categorized into < 32 years and ≥ 32 years based on median cumulative SHS exposure of years at home only. Subjects who were neither smokers nor second-hand smokers were considered as non-smokers.

### Validation of SHS exposure

To verify the participant's response that she was a cigarette smoker or had been exposed to SHS exposure reported on the questionnaire, we randomly selected 4 smokers, 23 second-hand smokers, and 44 non-smokers to provide one-spot urine specimens so that we could measure cotinine levels. Urinary cotinine was extracted by a liquid-liquid extraction method and measured by liquid chromatography tandem mass spectrometry (LC/MS/MS) equipped with a triple-quadruple mass spectrometer and TurboIonSpray™ (API 3000™, Applied Biosystems, Foster City, CA, USA) [30]. The urine specimen was co-spiked with cotinine-*d*_3 _as an internal standard. The detection limit for cotinine was 0.07 ng/ml. Urinary creatinine was measured by spectrophotometer (U-2000, Hitachi, Tokyo, Japan) with a wavelength set at 520 mm.

**Figure 1:**
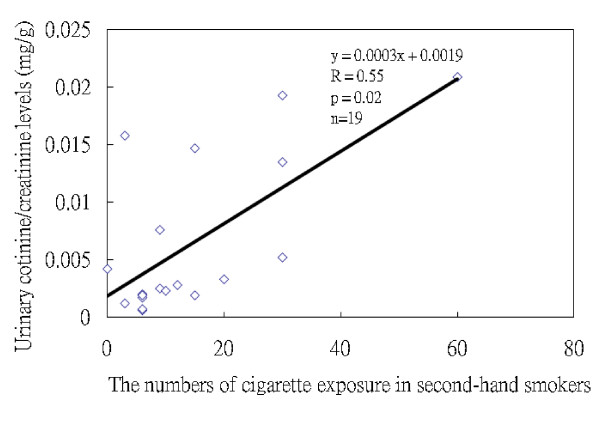
**Correlation between the urinary cotinine levels (mg/g creatinine) and the number of cigarettes they reported themselves as being exposed to SHS during the 3 days leading up to the collection of urine specimens**. (Spearman correlation coefficients, r = 0.55, *p *= 0.02, n = 19)

Since cotinine can only be used to test recent exposure to cigarette smoke, we could only use it to confirm the questions regarding the smoking of cigarettes and exposure to cigarettes in the past three days prior to the interview, when the urine specimens were collected. The mean urinary cotinine levels in the four active smokers were 0.79 ± 0.13 mg/g creatinine, significantly higher than that found for the 23 second-hand smokers (0.006 ± 0.006 mg/g creatinine) and 44 non-smokers (0.002 ± 0.002 mg/g creatinine) (both *p *< 0.01). The urinary cotinine levels in the second-hand smokers were also significantly higher than those in the non-smokers (*p *< 0.01) (Additional file 2). Nineteen of 23 second-hand smokers had information about the number of cigarettes they had been exposed to in the past three days prior to the interview. We found a high correlation between the number of cigarettes they reported being exposed to and the urinary cotinine levels (Spearman correlation coefficients, r = 0.55, *p *= 0.02, n = 19) (Figure 1), confirming the validity of our SHS questionnaire.

### Outcome measurements

#### Pulmonary function test

Pulmonary function measurement is the most important indicator of respiratory impairment in COPD [6]. Thus, after the face-to-face interview in the participant's home, the interviewers asked the participant to perform a pulmonary function test using a portable spirometer (Micro Direct Inc. MS03 MicroPlus, Rochester, England) for our community survey, which has been used by another study [31]. Spirometers were calibrated daily using a 3-L syringe. Forced expiratory volume in 1^st ^second (FEV_1_), forced vital capacity (FVC) and FEV_1_/FVC were recorded [1]. Each participant performed the pulmonary function tests at least three times. The highest of the scores were recorded. None of the participants had smoked, eaten, or used any bronchodilators within one hour prior to performing the pulmonary function tests. Allpulmonary function tests followed the guidelinesof the American Thoracic Society (ATS) [1]. To calculate the percent predicted values for lung function, we used predictive equations derived from Baldwin's formula for FVC and Berglund's formula for FEV_1 _[32,33].

#### Chronic bronchitis related health status

We used two disease classification systems to categorize chronic bronchitis-related health status in our data analysis (Additional file 1). First, we classified all study subjects into three disease groups based on (1) a physician's diagnosis of chronic bronchitis at least twice in year 1999 and on (2) the ATS criteria for chronic bronchitis (the presence of cough and/or sputum production during the majority of days for at least three consecutive months in the previous two or more successive years) [1]. The three disease groups included 33 chronic bronchitis sufferers (those who satisfied both criteria), 182 probably with chronic bronchitis (those who satisfied any one of criteria), and 205 with no chronic bronchitis (those who satisfied neither set of criteria).

Second, using pulmonary function test data (FEV_1 _and FVC), we categorized COPD into various levels of severity as described previously by the National Heart, Lung, and Blood Institute/World Health Organization GOLD criteria (Stages 0-IV) [6]. We classified 230 subjects as being Stage 0, meaning that lung function was normal (FEV_1_/FVC ≥ 70% and FEV_1 _≥ 80% predicted), 24 as being Stage I, meaning that airflow was mildly limited (FEV_1_/FVC<70% and FEV_1 _≥ 80% predicted), 41 as being Stage II, meaning that airflow was increasingly limited (FEV_1_/FVC<70% and 50% ≤ FEV_1_<80% predicted), 13 as being Stage III (FEV_1_/FVC<70% and 30% ≤ FEV_1_<50% predicted) and finally 4 as being Stage IV (FEV_1_/FVC<70% and FEV_1_<30% predicted), meaning that air flow was severely limited. We combined the Stage II to Stage IV as one group due to the small sample size. For the same reason, we moved those pulmonary function test scores ranging between FEV_1_/FVC ≥ 70% and 30% ≤ FEV_1 _< 80% into Stage I group. After combining these groups, 230 could be classified as being Stage 0 (renamed as No COPD), 129 as being Stage I (renamed as Mild COPD) and 58 as being Stage II-IV (renamed as Moderate COPD). Three subjects were excluded because of no pulmonary function test information.

### Statistical Analysis

Paired *t*-statistics and univariate conditional logistic regression were first used to compare the demographics of the 210 study cases and their age- and district-matched controls. After we further crosschecked using criteria set by the ATS into three disease groups and severity of COPD, χ^2 ^and Fisher's exact tests, or ANOVA statistics, when appropriate, were used to compare the differences of different variables. We used polytomous logistic regressions to elucidate the association between cigarette smoking (yes *vs*. no) and SHS exposure (yes *vs*. no), including cumulative SHS exposure categories (<32 and ≥ 32 years), and health status outcomes (based on physician diagnosis and ATS criteria, and spirometry by GOLD criteria) in three age periods and then combined these into a lifetime period, adjusting for age, height, educational level, cooking status, burning incense, and tea consumption. Statistical significance of trend in the adjusted odds ratios (AORs) across cigarette smoking and SHS exposure and different health status outcomes were calculated by categorizing variables and treating scored variables as continuous. Multiple linear regressions were used to investigate the relationship between the same individual variables and pulmonary function (FEV_1_, FVC and FEV_1_/FVC). In addition, we calculated the population attributable risk percentages (PAR%) of cigarette smoking and SHS exposure for chronic bronchitis and probable chronic bronchitis, also for severity of COPD [34]. The data was analyzed using the SAS statistical package; all *p*-values were two-sided.

## Results

### Characteristics of subjects on different health outcomes

Firstly, we had 210 study cases and 210 age- and district-matched controls (Additional file 1). There were no significant differences in the age, height, weight, education levels, and cigarette smoking between the two groups (data not shown). Only 3% of women had ever worked in a restaurant. The mean FEV_1 _levels (L) in the study cases and controls were 1.48 ± 0.42 and 1.64 ± 0.49 (*p *< 0.01), and the mean FVC levels (L) were 1.90 ± 0.53 and 2.00 ± 0.61 (*p *= 0.02) respectively.

Table 1 shows the demographic distributions of three disease groups, chronic bronchitis, probable chronic bronchitis and no chronic bronchitis groups. Weight and BMI were significantly different among these three disease groups (*p *= 0.01). Those with chronic bronchitis had significantly lower lung function in FEV_1_, FVC and FEV_1_/FVC than either of the other two groups. In addition, the smoking rate in the group of chronic bronchitis was 24.2%, significantly higher than the other two groups (5.8 and 3.9%, *p *< 0.01).

**Table 1:** Demographic characteristics of chronic bronchitis, probable chronic bronchitis, and no chronic bronchitis according to physician diagnosis and ATS criteria (n = 420)^a^

Variables	Chronic bronchitis (n = 33)	Probable chronic bronchitis (n = 182)	No chronic bronchitis (n = 205)	*p*-value
Mean (SD)				

Age (yrs)	65.2 (12.3)	64.6 (9.1)	64.5 (9.7)	0.94
Height (cm)	154.5 (4.3)	155.5 (5.7)	155.2 (4.8)	0.59
Weight (kg)	54.0 (10.0)	57.7 (7.5)	58.5 (7.5)	0.01
BMI	22.6 (3.9)	23.9 (3.0)	24.3 (3.1)	0.01
Pulmonary function test				
FEV_1 _(L)	1.33 (0.52)^a^	1.51 (0.40)	1.64 (0.48)	<0.01
FVC (L)	1.74 (0.58)^a^	1.94 (0.52)	2.00 (0.62)	0.05
FEV_1_/FVC (%)	76.1 (17.1)^a^	78.9 (15.0)	83.8 (15.5)	<0.01

N (%)				

Education levels				
≥ junior high school	5 (15.2)	32 (17.6)	34 (16.6)	0.67
primary school	16 (48.5)	93 (51.1)	117 (57.1)	
illiteracy	12 (36.4)	57 (31.3)	54 (26.3)	
Cigarette smoking				
no	25 (75.8)	172 (94.5)	197 (96.1)	<0.01^b^
yes	8 (24.2)	10 (5.5)	8 (3.9)	
Alcohol consumption				
no	33 (100)	176 (96.7)	200 (97.6)	0.14^b^
yes	0	6 (3.4)	5 (2.5)	
Tea consumption				
no	31 (93.9)	152 (83.5)	164 (80.0)	0.13^b^
yes	2 (6.1)	30 (16.5)	41 (20.0)	
Burning incense				
no	14 (42.4)	81 (44.5)	89 (43.4)	0.96
yes	19 (57.6)	101 (55.5)	116 (56.6)	
Cooking status				
no	2 (6.1)	9 (5.0)	6 (2.9)	0.50^b^
Yes	31 (93.9)	173 (95.1)	199 (97.1)	

^a^Three subjects did not have complete lung function test information

^b^Fisher's exact test.

### Impact of SHS exposure on health status based on physician diagnosis and ATS criteria

As shown in Table 2, we analyzed the three disease groups by whether or not they smoked or were exposed to SHS, further categorized into less than 32 years and more than 32 years exposure in their lifetime. Smokers and second-hand smokers were 24.81-fold (95% CI: 5.78-106.38) and 3.65-fold (95% CI: 1.19-11.26) more likely to have chronic bronchitis than the non-smokers. In addition, those with SHS exposure under 32 years had a significantly greater risk (AOR = 4.26, 95%CI: 1.29-14.09) of having chronic bronchitis than non-smokers, though this was not found in those with SHS exposure over 32 years (AOR = 3.02; 95%CI: 0.85-10.70) due to small sample size. For probable chronic bronchitis, we also noted a significant increasing trend of risk by years of SHS exposure compared to those not exposed to SHS (trend test, *p *= 0.01). The PAR% for chronic bronchitis was 23.2% and 47.3% in smokers and those exposed to SHS respectively.

**Table 2:** Relationships between cigarette smoking and SHS exposure in lifetime status and chronic bronchitis and probable chronic bronchitis according to physician diagnosis and ATS criteria.

Variables	No chronic bronchitis (n = 205)	Probable chronic bronchitis (n = 182)			Chronic bronchitis (n = 33)		
			
	N (%)	N (%)	**AOR (95%CI)**^a^	PAR (%)	N (%)	AOR (95%CI)^a^	PAR (%)
*Cigarette smoking/SHS status*							
Non-smoker	76 (37.1)	52 (28.6)	1		4 (12.1)	1	
Second-hand smoker	121 (59.0)	120 (65.9)	1.57 (1.00-2.45)	23.8	21 (63.6)	3.65 (1.19-11.26)^c^	47.3
Smoker	8 (3.9)	10 (5.5)	1.99 (0.72-5.48)	2.6	8 (24.2)	24.81 (5.78-106.38)^c^	23.2

*Cigarette smoking and* *cumulative SHS exposure (year)*							
Non-smoker	76 (37.1)	52 (28.6)	1		4 (12.1)	1	
Second-hand smoker							
1< SHS < 32	67 (32.7)	53 (29.1)	1.24 (0.74-2.10)		13 (39.4)	4.26 (1.29-14.09)^c^	
SHS ≥ 32	54 (26.3)	67 (36.8)	1.93 (1.16-3.23)^b^		8 (24.2)	3.02 (0.85-10.70)^b,c^	
Smoker	8 (3.9)	10 (5.5)	2.00 (0.73-5.51)		8 (24.2)	24.74 (5.77-106.15)	

^a^AOR: Adjusting for age, secondhand smoke status, height, education level, cooking status, burning incense and tea consumption.

^b^Trend test from non-SHS, 1<SHS<32, to SHS ≧ 32 years in probable chronic bronchitis: *p *= 0.01; and in chronic bronchitis: *p *= 0.12.

^c^Trend test from no chronic bronchitis, probable chronic bronchitis, to chronic bronchitis in secondhand smokers: *p *= 0.01 (*p *= 0.05 in 1<SHS<32 years and *p *= 0.01 in SHS ≧ 32 years); and in smoker: *p *< 0.01.

### Impact of SHS exposure on health status based on spirometry by GOLD criteria

Table 3 shows smokers and second-hand smokers were 6.49-fold (95% CI: 1.61-26.25) and 3.84-fold (95% CI: 1.72-8.60) more likely to have moderate COPD than the non-smokers. In addition, those with SHS exposure under 32 years (AOR = 3.31, 95%CI: 1.36-8.06) and over 32 years (AOR = 4.43, 95%CI: 1.85-10.60) had significantly greater risks of having moderate COPD than non-smokers. We also noted a significant increasing trend of risk of having mild COPD and moderate COPD by years of SHS exposure, compared to those not exposed to SHS (trend test, *p *= 0.01 and < 0.01 respectively). Smokers, making up a small percentage of our study population, had a PAR% of 8.9% and 7.3% for mild COPD and moderate COPD, whereas second-hand smokers, making up 62% of our study population, had a PAR% of 26.5% and 56.2% for mild and moderate COPD.

**Table 3:** Relationships between cigarette smoking and SHS exposure in lifetime status and the severity of COPD according to GOLD criteria.

Variables	No COPD (n = 230)	Mild COPD (n = 129)			Moderate COPD (n = 58)		
			
	N (%)	N (%)	AOR (95%CI)^a^	PAR (%)	N (%)	AOR (95%CI)^a^	PAR (%)
*Cigarette smoking/SHS status*							
Non-smoker	90 (39.1)	32 (24.8)	1		9 (15.5)	1	
Second-hand smoker	133 (57.8)	83 (64.3)	1.76 (1.06-2.93)	26.5	44 (75.9)	3.84 (1.72-8.60)^c^	56.2
Smoker	7 (3.0)	14 (10.9)	7.01 (2.52-19.51)	8.9	5 (8.6)	6.49 (1.61-26.15)^c^	7.3

*Cigarette smoking and cumulative SHS exposure (year)*							
Non-smoker	90 (39.1)	32 (24.8)	1		9 (15.5)	1	
Second-hand smoker							
1 < SHS < 32	73 (31.7)	38 (29.5)	1.48 (0.82-2.68)		20 (34.5)	3.31 (1.36-8.06)^c^	
SHS ≥ 32	60 (26.1)	45 (34.9)	2.05 (1.16-3.64)^b^		24 (41.4)	4.43 (1.85-10.60)^b,c^	
Smoker	7 (3.0)	14 (10.9)	7.04 (2.53-19.61)		5 (8.6)	6.51 (1.61-26.32)	

^a^AOR: Adjusting for age, secondhand smoke status, height, education level, cooking status, burning incense and tea consumption.

^b^Trend test from Non-SHS, 1<SHS<32, to SHS ≧ 32 years in Mild COPD: *p *= 0.01; and in Moderate COPD: *p *< 0.01.

^c^Trend test from No COPD, Mild COPD, to Moderate COPD in second-hand smoker: *p *< 0.01 (*p *< 0.01 in 1<SHS<32 years and SHS ≧ 32 years); and in smoker: *p *< 0.01.

### Impact of SHS exposure on pulmonary function status

Using FEV_1_, FVC or FEV_1_/FVC ratio as an outcome indicator, we found there was a more significant worsening in mean FEV_1 _values in smokers (246 mL, *p *< 0.01) and second-hand smokers (104 mL, *p *= 0.01) than in non-smokers, after adjusting for other covariates (data not shown). In addition, mean FEV_1 _values significantly decreased 113 mL (*p *= 0.01) and 95 mL (*p *= 0.04) in subjects exposed to SHS ≥ 32 years and < 32 years respectively, compared to those in non-smokers, after adjusting for cigarette smoking and other covariates (Table 4). Similar results were found when we used FEV_1_/FVC ratio as an indicator (Table 4). No significant effects of cigarette smoking and SHS exposure on FVC measurements were observed (data not shown).

**Table 4:** Predictors of absolute FEV_1 _level and FEV_1_/FVC (%): in a multiple linear regression model^a^

Variables			Adjusted analysis			Adjusted analysis	
					
	N	FEV1 (L) (mean ± SD)	β(SE)	*p*-value	FEV1/FVC (%)(mean ± SD)	β(SE)	*p*-value
Age	417	1.56 ± 0.46	-0.022 (0.002)	<0.01	81.10 ± 15.59	-0.134 (0.090)	0.14
Height	417		0.021 (0.004)	<0.01		0.421 (0.146)	<0.01
Education levels							
≥junior high school	71	1.80 ± 0.41	1		80.94 ± 14.79	1	
primary school	225	1.59 ± 0.46	-0.044 (0.053)	0.41	81.04 ± 15.79	1.820 (2.142)	0.4
illiteracy	121	1.37 ± 0.40	-0.079 (0.063)	0.21	81.32 ± 15.79	3.068 (2.554)	0.23
Cigarette smoking and cumulative SHS exposure year							
Non-smoker	131	1.60 ± 0.41	1		86.83 ± 12.57	1	
Second-hand smoker							
1 < SHS < 32	131	1.63 ± 0.47	-0.095 (0.046)	0.04	80.70 ± 14.75	-5.807 (1.900)	<0.01
SHS ≥ 32	129	1.51 ± 0.47	-0.113 (0.046)	0.01	77.07 ± 16.86	-9.436 (1.869)	<0.01
Smoker	26	1.29 ± 0.50	-0.246 (0.079)	<0.01	74.24 ± 18.18	-11.592 (3.226)	<0.01
Burning incense							
no	182	1.63 ± 0.47	1		81.70 ± 15.57	1	
yes	235	1.51 ± 0.44	-0.091 (0.037)	0.02	80.64 ± 15.62	-1.654 (1.523)	0.28
Cooking status							
no	17	1.56 ± 0.48	1		83.80 ± 15.69	1	
yes	400	1.56 ± 0.46	0.012 (0.094)	0.9	80.99 ± 15.59	-2.243 (3.805)	0.56

^a^Three subjects did not have complete lung function test information.

## Discussion

### Main findings of this study

This study indicates that, besides cigarette smoking, SHS exposure significantly increases the risk of chronic bronchitis in non-smoking Taiwanese women by using different outcome indicators, including physician-diagnosis/ATS criteria, the severity of COPD [6] and pulmonary function (FEV_1 _or FEV_1_/FVC ratio) impairment. We also used the urinary cotinine levels for the validation of self-report to SHS exposure. These results were also seen when exposure assessment was based on duration of SHS exposure at home, rather than household-reported cigarette smoke use alone.

Our results of increased risk of chronic bronchitis with SHS exposure in women concur with some previous studies [24,26,27]. However, some of the studies have shown mixed results (Additional file 3). The definitions of COPD/chronic bronchitis may be one of the reasons. We used two sets of disease classification of chronic bronchitis concurrently, to reduce the possibility of misclassification of our surveyed subjects and illustrate the effect of chronic bronchitis more clearly. One case-control study conducted by Kalandidi and his colleagues found that women married to smokers and exposed to ≤ 1 pack/day were at 2.5 times (95% CI: 1.3-5.0) a greater risk of having COPD than those married to non-smokers, but did not find a similar increase in women whose spouses smoked > 1 pack/day [24]. Simoni and his colleagues, performing a similar study, found female spouses of smokers to be at 2.24 times (95%CI: 1.40-3.58) more risk to have obstructive lung disease than those married to non-smokers. Unfortunately, this study population covered asthma or emphysema disease and this might reflect the complex nature of the disease [27]. In a longitudinal study, Sandler and his colleagues followed 14,783 healthy subjects exposed to SHS for 12 years and found that the estimated relative risk (RR) of death for emphysema or bronchitis was 5.65 (95% CI: 1.19-26.8); this result may be confounded by the small number of deaths (13) in that study [26].

A dose-response relationship was noted between duration of SHS exposure at home by the cut-point of 32 SHS exposure years and COPD severity based on spirometry, although it was present based on the criteria of physician diagnosis and crosschecked with ATS criteria. The probable reason is: the category of disease phenotype by ATS criteria, which was subjective, was not as accurate as by pulmonary function tests, which was objective. One study reported that only those with long duration of SHS exposure (42 years or more at home) were at increased risk of COPD (AORs:1.55; 95%CI:1.09-2.21) [17]. Thus, our findings add additional information about the association of cumulative SHS exposure with COPD risk in non-smokers.

Cotinine in urine is widely used as a biomarker of SHS exposure in epidemiology studies [35]. Although our study relied on self-reports of SHS exposure, we measured urinary cotinine levels in a subset of study subjects to verify the questionnaire data, as similarly done with other studies [23,27,28]. We found the urinary cotinine levels in smokers, second-hand smokers and non-smokers to agree well with a report that urinary excretion of the nicotine metabolites in smokers is approximately one hundred times higher than that observed in second-hand smokers and non-smokers [36]. In our study, the more exposure to SHS our second-hand smokers self-reported per day, the higher their cotinine levels, confirming the validity of response to the questionnaire item. This agreement has been found by others [37-39]. Eisner and his colleagues reported the first study indicating that the highest urine cotinine tertile was associated with poorer COPD severity scores both in cross-sectional or 1-year follow-up study [18], which adds substantive additional evidence that SHS exposure is deleterious for patients with COPD by objective measures of urinary cotinine levels.

### What this study adds

PAR% has been used to judge priorities for public health action [34,40]. Because there is a high prevalence of cigarette smoking in Caucasian men and women, several studies in those countries have considered the PAR% with regard to smoking and COPD [8,41,42]. The smoking attributable fraction of COPD mortality in the United States has been reported to be 69.4% for women. In that same time period, smoking was prevalent in 28.3-31.6% of the women and 31.8-40.6% of the men in the United States [8]. In Taiwan, the gap in smoking prevalence between women and men (3-4% *vs*. 55-60%) is much wider, so using PAR% would be helpful to study the contribution of SHS exposure to the risk of chronic bronchitis. Eisner and his colleagues reported that the PAR% was eleven percent for the highest quartile of home SHS exposure and seven percent for work exposure, though it is not known how they calculated these percentages [17].

The results of the above studies are, except for age, based on a single risk factor without regard to other factors [27,41]. Using a multivariate model to calculate summary PAR% [34], we found although the AORs for chronic bronchitis for smokers is high, the prevalence of cigarette smoking among women is low. This made the PAR% for chronic bronchitis caused by cigarette smoking to be quite low (23.2%) (Table 2). In contrast, we found the AORs for chronic bronchitis showed that women exposed to SHS were 3.65 times more likely than those not exposed to SHS to have chronic bronchitis. This risk combined with a very high prevalence rate for exposure to SHS yielded a 47.3 PAR%, higher than the report from Simoni and his colleagues (adjusted PAR% = 12%) [27], making SHS a more important public health issue in non-smoking women. Similar results of PAR% occurred when we analyzed the SHS-attributable fraction of COPD severity (Table 3). The above results suggest that SHS exposure may be the underlying reason for the high prevalence of chronic bronchitis among non-smoking Taiwanese women. As shown in our female population, who smoke relatively rarely (3-4%), but live with high male smoking prevalence (55-62%) [9], this provides a good opportunity to add information on poorly investigated respiratory disease effects of lifetime SHS exposure in Taiwanese non-smoking women.

### Limitations of this study

Our study has some limitations. First, the prevalence rate for chronic bronchitis in women was relatively low, probably because we required that each woman must be diagnosed as having chronic bronchitis at least twice that year in order to be included as an experimental case from our national claims data. This would cause an underestimation. Second, we have only randomly selected about 17% study cases for interview to date and the response rates in the groups of study cases and controls were about sixty-seven percent, which was not very high. This might increase the likelihood of selection bias; however, the age of responders and non-responders was comparable, and unlikely to bias our results. In addition, subjects were blinded to the purpose of our study, so their concern about lung function with their exposure or disease outcome should not cause bias in our study. Third, in this study, there was probably some recall bias regarding estimates of lifetime exposure status. Still, the misclassification of exposure is likely to be random, which would have null effect on our results. Regardless, we were still able to find significant risk, suggesting that this bias might not exert much influence on our results.

## Conclusion

In conclusion, we found a dose-response trend on the effect of lifetime exposure to SHS and risk for chronic bronchitis, with SHS exposure accounting for 47.3% of the attributable risks of having chronic bronchitis. Our findings add additional evidence to the growing body of knowledge supporting the great need for health policies to ensure that a smoke-free environment is created and maintained for the public to decrease the risk of adverse health consequences by non-smokers exposed to SHS.

## Competing interests

The authors declare that they have no competing interests.

## Authors' contributions

WC was responsible for data collection, statistical analysis, data interpretation, and wrote the first draft of the manuscript. FN, CI, HJ and CS contributed the clinical data collection, data interpretation and critical revision of the manuscript. WK was responsible for urinary cotinine and creatinine measurement for quality assurance and critical revision of the manuscript. LC contributed the statistical analysis, data interpretation and critical revision of the manuscript. HC and LC co-wrote the study design, and contributed to the statistical analysis, data interpretation and critical revision of the manuscript. CD was responsible for study supervision and critical revision of the manuscript. WM created the original study design, and was responsible for protocol development, survey design, data interpretation and critical revision of the manuscript. All authors read and approved the final version.

## Pre-publication history

The pre-publication history for this paper can be accessed here:

http://www.biomedcentral.com/1471-2458/10/44/prepub

## Supplementary Material

Additional file 1 **Appendix 1**. The flow-chart of the study design. Click here for file

Additional file 2 **Appendix 2**. The distribution of natural log-transformed urine cotinine/creatinine levels among the groups of smokers (n = 4), second-hand smokers (n = 23), and non-smokers (n = 44). Click here for file

Additional file 3 **Appendix 3**. Summary of epidemiologic studies on the relationship between SHS exposure and the risk of chronic obstructive pulmonary disease (COPD) among women. Click here for file
