# Supplementary material for: Second-hand smoke and chronic bronchitis in Taiwanese women: a health-care based study
Source: BMC Public Health. 2010 Jan 28;10:44. doi: 10.1186/1471-2458-10-44 (PMC2841674; doi:10.1186/1471-2458-10-44)
Supplement: Additional file 1 — Appendix 1. The flow-chart of the study design. [file 1471-2458-10-44-S1.DOC]

# **Additional files**

Taiwan National Health Insurance Bureau in year 1999

Women who were 40 years old and over

and had lived in Kaohsiung city for 5 years

**Study Case**

Criteria: Diagnosed with definite or suspected chronic bronchitis (ICD-9 code: 491) at least twice by physicians in that year and had no other pulmonary-associated diseases (n=1,846)

**Study Control**

Criteria: Diagnosed with traffic accident (ICD-9 code: E800-E848) or acute gastroenteritis (ICD-9 code: 008.8; 009.1; 558.3; 558.9) and without any pulmonary-associated diseases, including chronic bronchitis (n=4,624)

About one-third study cases (n=600) and matching controls (n=1,200) were recruited for interview between 2000 and 2005, including questionnaire, pulmonary function tests, and the collection of the urine specimens

For complete interview: we had **210 study cases and 210 study controls**

**Two outcome indicators**

**Based on physician diagnosis and ATS criteria**

Chronic bronchitis: Diagnosed by physicians and satisfied by ATS criteria (n=33)

Probable chronic bronchitis: Diagnosed by physicians but not yet satisfied by ATS criteria (n=182)

No chronic bronchitis: Neither diagnosed as chronic bronchitis (n=205)

**Based on spirometry by GOLD criteria (severity)**

COPD GOLD IV: FEV_1_/FVC < 0.7 and FEV_1_ < 30% predicted

COPD GOLD III: FEV_1_/FVC < 0.7 and 30% ≤ FEV_1_ < 50% predicted

COPD GOLD II: FEV_1_/FVC < 0.7 and 50% ≤ FEV_1_ < 80% predicted

COPD GOLD I: FEV_1_/FVC < 0.7 and FEV_1_ ≥ 80% predicted

No COPD: FEV_1_/FVC ≥ 0.7 and FEV_1_ ≥ 80%

**Specimens: urine**

**Subgroup (n=71)**

Analytic method: LC/MS/MS

Result: Cotinine levels

Statistic analysis

**Figure 1** The flow chart of the study design.
