# Supplementary material for: Second-hand smoke and chronic bronchitis in Taiwanese women: a health-care based study
Source: BMC Public Health. 2010 Jan 28;10:44. doi: 10.1186/1471-2458-10-44 (PMC2841674; doi:10.1186/1471-2458-10-44)
Supplement: Additional file 2 — Appendix 2. The distribution of natural log-transformed urine cotinine/creatinine levels among the groups of smokers (n = 4), second-hand smokers (n = 23), and non-smokers (n = 44). [file 1471-2458-10-44-S2.DOC]

# **Additional files**


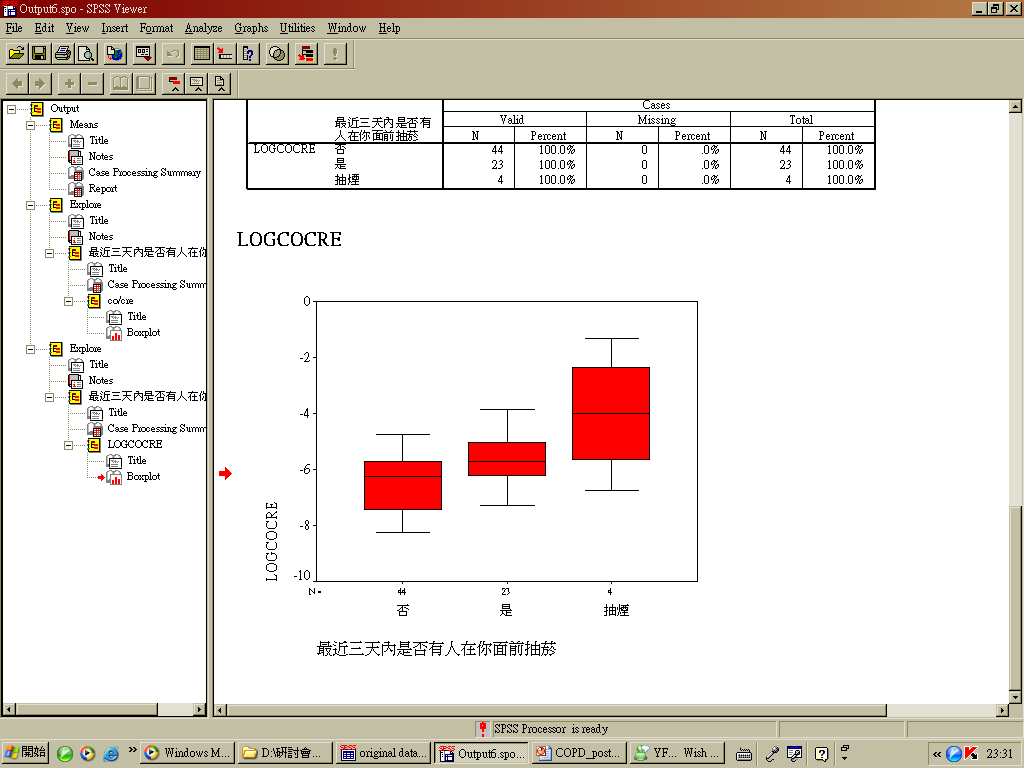


**Non-smokers Secondhand smokers Smokers**

**Figure 2** The distribution of natural log-transformed urine cotinine/creatinine levels (mg/g creatinine) among the groups of smokers (n=4), secondhand smokers (n=23), and non-smokers (n=44).
