# Supplementary material for: Second-hand smoke and chronic bronchitis in Taiwanese women: a health-care based study
Source: BMC Public Health. 2010 Jan 28;10:44. doi: 10.1186/1471-2458-10-44 (PMC2841674; doi:10.1186/1471-2458-10-44)
Supplement: Additional file 3 — Appendix 3. Summary of epidemiologic studies on the relationship between SHS exposure and the risk of chronic obstructive pulmonary disease (COPD) among women. [file 1471-2458-10-44-S3.DOC]

**Additional file 3 -** Summary of epidemiologic studies on the relationship between SHS exposure and the risk of chronic obstructive pulmonary disease (COPD) among women.

| Study | Subjects | SHS exposure assessment | Exposure validation | Results |
| --- | --- | --- | --- | --- |
| **Case-control study** | |  |  |  |
| 1986, UK  1982/7-1983/8  [25] | 17 chronic bronchitis  318 controls | Place evaluated: home+work+other  Qualitative: spouse’s smoking and others  Quantitative: combined index score 2-4 and 5-12^*^ | No | OR was 1.22 for spouse’s smoking, and for combined index of 2-4 and 5-12 are 1.05 and 1.03, but all were non-significant |
| 1987, Greece  1982-1983  [24] | 103 COPD  179 controls | Place evaluated: home  Qualitative: spouse’s smoking  Quantitative: packs/day & life-long consumption | No | AORs was 2.5 (95% CI = 1.3-5.0) and 1.5 (95% CI = 0.8-2.7) for spouse’s smoking ≤1 and >1 packs/day, but not found significant in life-long consumption |
| **Cross-sectional study** | |  |  |  |
| 2000, Italian  1997-1998  [23] | 50 chronic obstructive pulmonary disease of 1,938 women | Place evaluated: home  Qualitative: spouse’s smoking  Quantitative: - | Salivary cotinine | AORs is 1.80 (95%CI=0.88-3.47) |
| 2007, Italian  [27] | 165 obstructive lung disease of 2,195 women | Place evaluated: home+work  Qualitative: spouse’s smoking  Quantitative: - | Salivary cotinine | AORs is 2.24 (95%CI=1.40-3.58). |
| **Longitudinal study** | |  |  |  |
| 1989, USA  1963-1975  [26] | 14,873 subjects followed-up for 12 yrs  13 COPD deaths | Place evaluated: home  Qualitative: household member smoking  Quantitative: - | No | RR was 5.65 (95% CI= 1.19-26.8) for the death of emphysema/bronchitis |
| 2003, USA  1960-1998  [22] | 25,942 subjects followed-up for 39 yrs  128 COPD deaths | Place evaluated: home  Qualitative: spouse’s smoking  Quantitative: - | No | RR was 1.13 (95% CI = 0.80-1.58) for the death of COPD |
| 2005, European  1993-1998  [28] | 123,479 subjects followed-up for 7 yrs  14 COPD deaths | Place evaluated: home+work  Qualitative: household member smoking  Quantitative: - | Plasma cotinine | Hazard ratio was 1.15 (95% CI= 0.71-1.86); AORs was 1.46 (95% CI = 0.76-2.80) in the nested case-control study |
|  |  |  |  |  |
| **Community case-control study** | |  |  |  |
| 2009, In our study  Taiwan  2000-2005 | 33 chronic bronchitis  182 probable cases  205 no chronic bronchitis | Place evaluated: home+work  Qualitative: household member smoking  Quantitative: duration (years) | Urinary cotinine | AORs in lifetime SHS exposure was 3.65-fold risk (95% CI = 1.19-11.26) in chronic bronchitis. The more the exposure years of SHS, the risk of severity COPD increased in trend (*p* < 0.01) |

^*^ Based on sum of 0=not at all; 1=little; 2=average; 3=a lot for passive smoke exposure at home, at work, during travel and during leisure.
